# MALDI-TOF Mass Spectrometry: A Powerful Tool for Clinical Microbiology at Hôpital Principal de Dakar, Senegal (West Africa)

**DOI:** 10.1371/journal.pone.0145889

**Published:** 2015-12-30

**Authors:** Cheikh I. Lo, Bécaye Fall, Bissoume Sambe-Ba, Silman Diawara, Mamadou W. Gueye, Oleg Mediannikov, Cheikh Sokhna, Ngor Faye, Yaya Diemé, Boubacar Wade, Didier Raoult, Florence Fenollar

**Affiliations:** 1 Unité de Recherche sur les Maladies Infectieuses et Tropicales Emergentes, UM63, CNRS7278, IRD198, InsermU1095, Institut Hospitalo-Universitaire Méditerranée-Infection, Aix-Marseille Université, Marseille, France and Dakar, Senegal; 2 Hôpital Principal de Dakar, Dakar, Senegal; 3 Université Cheikh Anta Diop, Dakar, Senegal; University of Glasgow, UNITED KINGDOM

## Abstract

Our team in Europe has developed the routine clinical laboratory identification of microorganisms by matrix-assisted laser desorption ionization time-of-flight (MALDI-TOF) mass spectrometry (MS). To evaluate the utility of MALDI-TOF MS in tropical Africa in collaboration with local teams, we installed an apparatus in the Hôpital Principal de Dakar (Senegal), performed routine identification of isolates, and confirmed or completed their identification in France. In the case of discordance or a lack of identification, molecular biology was performed. Overall, 153/191 (80.1%) and 174/191 (91.1%) isolates yielded an accurate and concordant identification for the species and genus, respectively, with the 2 different MALDI-TOF MSs in Dakar and Marseille. The 10 most common bacteria, representing 94.2% of all bacteria routinely identified in the laboratory in Dakar (*Escherichia coli*, *Klebsiella pneumoniae*, *Streptococcus agalactiae*, *Acinetobacter baumannii*, *Pseudomonas aeruginosa*, *Staphylococcus aureus*, *Staphylococcus haemolyticus*, *Enterobacter cloacae*, *Enterococcus faecalis*, and *Staphylococcus epidermidis*) were accurately identified with the MALDI-TOF MS in Dakar. The most frequent misidentification in Dakar was at the species level for *Achromobacter xylosoxidans*, which was inaccurately identified as *Achromobacter denitrificans*, and the bacteria absent from the database, such as *Exiguobacterium aurientacum* or *Kytococcus schroeteri*, could not be identified. A few difficulties were observed with MALDI-TOF MS for *Bacillus* sp. or oral streptococci. 16S rRNA sequencing identified a novel bacterium, “*Necropsobacter massiliensis*.” The robust identification of microorganisms by MALDI-TOF MS in Dakar and Marseille demonstrates that MALDI-TOF MS can be used as a first-line tool in clinical microbiology laboratories in tropical countries.

## Introduction

The routine identification of bacteria and fungi by matrix-assisted laser desorption ionization time-of-flight (MALDI-TOF) mass spectrometry (MS) has become a true revolution in clinical microbiology laboratories [1,2]. Indeed, this technique is a powerful and robust tool for accurately identifying bacteria and fungi in less than 1 hour without a priori knowledge of the type of microorganisms; it is also more cost-effective than current phenotypic testing methods, despite the initial cost of the instrument and the maintenance costs, and perhaps most importantly, it is easy to use [1,2]. The rapid and accurate identification of microorganisms is necessary for the management of infectious diseases, particularly for choosing effective antibiotic therapies, reducing costs, and shortening hospital stays. MALDI-TOF MS has already become a first-line tool for routine microbial identification in many laboratories in Europe, and its use has become widespread in other areas, including Asia and America [2–7].

Since 2009, our laboratory in Marseille, France, has been a pioneer in the identification of bacteria using MALDI-TOF MS in the clinical microbiology laboratory [5]. Thus, for 6 years, the identification of bacteria has been routinely performed with our MALDI-TOF MS platform, and our database has undergone continuous revision [5,6]. Based on our extensive experience, we aimed to perform routine identification in a clinical microbiology laboratory in tropical Africa. Bacterial identification in Africa is challenging because conventional phenotypic methods require many reagents with specific storage conditions and shelf lives, in addition to higher consumable costs. Thus optimal conditions for bacterial identification are often lacking, and misidentification is prevalent. In addition, in the case of misidentification, molecular methods that are complementary for microbial identification cannot be performed due not only to complicated procedures but also the need for specific materials, reagents, and automates with very high cost. We believe that MALDI-TOF MS can be a great alternative for bacterial and fungal identification in Africa, when electricity is continuously available as well as air conditioning. Thus, we installed a MALDI-TOF MS platform in the Hôpital Principal de Dakar in Senegal [8].

Herein, we evaluated the potential for routine bacterial and fungal identification using MALDI-TOF MS in a clinical microbiology laboratory in Africa.

## Materials and Methods

### Isolates

Two hundred clinical isolates obtained by conventional culture procedures in the clinical microbiology laboratory of the Hôpital Principal de Dakar (Senegal) and tested for identification using MALDI-TOF MS (VITEK^®^ MS-RUO) were sent to the clinical microbiology laboratory of the University Hospital in Marseille to confirm or complete the identification performed in Africa using another mass spectrometer (MicroFlex LT, Bruker Daltonik). As each MALDI-TOF system has been regulatory approved, is commercialized, and currently used for the routine bacterial and fungal identification in clinical microbiology laboratories in several European countries and in USA, we speculate that when the identifications were concordant, they were accurate [9,10].

When isolates’ identifications had failed (incomplete or lack of identification) or were discordant between Dakar and Marseille, the isolates were systematically tested with an appropriate molecular biology method based on the sequencing of 16S rRNA, 18S rRNA, or *rpoB* sequences. In addition, for the *Bacillus cereus* group, additional phenotypic tests were performed to differentiate *Bacillus cereus* from *Bacillus anthracis* and *Bacillus thuringiensis*, as previously reported [11]. All the isolates were transported from Senegal to France in Portagerm Amies Agar swab transport tubes (bioMérieux) at room temperature.

### MALDI-TOF MS analysis in Dakar

MALDI-TOF MS analysis was performed in Dakar with a VITEK^®^ MS-RUO version 1.0 (bioMérieux). Each isolated colony was picked and placed in a single well of a disposable, barcode-labeled target slide (VITEK MS-DS, bioMérieux) using a 1-μl plastic loop. Then, each colony was covered with 1.0 μl of a saturated solution of alpha-cyano-4-hydroxycinnamic acid matrix (VITEK MS-CHCA, bioMérieux) and air-dried. Two spots were systematically created for each colony. For each assay, a strain of *Escherichia coli* (Lyfocults *E*. *coli* ATCC # 8739, bioMérieux) was also analyzed for quality control. Indeed, this *E*. *coli* reference strain was used for instrument calibration but also as a positive control.

Analyses of the obtained spectra were performed using the Saramis database version 4.0 (bioMérieux). The Saramis software color-codes identification results (by default) according to confidence levels as follows: 99.9%, dark green; 99.8% to 90.0%, light green; 89.9% to 85.0%, yellow; and 84.9% to 70.0%, white. Confidence levels between 70.0% and 99.9% were considered correct identification at the genus and species levels.

### MALDI-TOF MS analysis in Marseille

MALDI-TOF MS analysis was performed with a MicroFlex LT mass spectrometer (Bruker Daltonik) as reported [5,6,12]. Each isolated colony was deposited on a MALDI-TOF MS target Microflex (Bruker Daltonik) as above. Then, each colony was overlaid with 2 μL of matrix solution (saturated solution of alpha-cyano-4-hydroxycinnamic acid in 50% acetonitrile and 2.5% tri-fluoracetic-acid), and the matrix-sample was crystallized by air-drying at room temperature, as previously described [5,6,12]. Two spots were systematically created for each colony. For each assay, a strain of *Escherichia coli* (DH5 alpha, Bruker Daltonik) was also analyzed for quality control.

The analyses of the obtained spectra were performed using our personal database [5,6], the Bruker database updated with a laboratory collection of spectra from clinical isolates identified using molecular sequencing (primarily 16S rRNA sequencing) [6].

The criteria for identification were previously reported [5]. An isolate was considered correctly identified by MALDI-TOF MS if both spectra had a score ≥1.9 for species identification or ≥1.7 for genus identification [5].

Finally, positive controls are used in a routine manner for both MALDI-TOF MS systems. Besides, colonies are systematically tested in duplicate. The *E*. *coli* positive controls must be correctly identified and a same identification for the duplicate spots of each colony must be obtained using the score of each software package in order to conclude to the identification of microorganism.

### Identification by molecular biology methods

Molecular analyses using PCR and sequencing were performed targeting 16S ribosomal RNA (rRNA), 18S rRNA, or *rpoB* sequences, as previously described [13–16]. DNA from isolates was extracted using the MagNA Pure LC kit DNA isolation kit III with the EZ-1 biorobot (Qiagen, Hilden, Germany) according to the manufacturer’s instructions. All the primers used for PCR and the sequencing of 16S rRNA, 18S rRNA, and *rpoB* targets are summarized in Table 1 [13–19]. PCR products were purified using the PCR kit Nucleofast 96 (Macherey-Nagel, Hoerdt, France), and sequencing was performed with the Big Dye Terminator, version 1.1 sequencing kit (Perkin-Elmer, Coignieres, France) according to the manufacturers’ instructions. Products of the sequencing reaction were purified, and sequences were analyzed on an ABI PRISM 3130X Genetic Analyzer (Applied Biosystems, California, USA) [13,13,20,21]. The sequences were assembled and amended with CodonCode Aligner v4.1.1 software (CodonCode Corporation, USA). Then, a correct consensus sequence was saved and compared with the GenBank database using the BLAST software (http://blast.ncbi.nlm.nih.gov/Blast.cgi). An isolate was correctly identified when it yielded > 98.7% sequence identity for the 16S rRNA sequence and > 97% sequence identity for the *rpoB* sequence with the closest bacterial species sequence in GenBank [13,15,16,22–24].

**Table 1:** List of all primers used for PCR and sequencing analyses in this study.

Targeted sequences	Primers	Sequences (5’– 3’)	Method	References
16S rRNA	Fd1	AGA GTT TGA TCC TGG CTC AG	PCR	[21]
	Rp2	ACG GCT ACC TTG TTA CGA CTT	PCR	[21]
	536F	CAG CAG CCG CGG TAA TAC	Sequencing	[21]
	536R	GTA TTA CCG CGG CTG CTG	Sequencing	[21]
	800F	ATT AGA TAC CCT GGT AG	Sequencing	[21]
	800R	CTA CCA GGG TAT CTA AT	Sequencing	[21]
	1050F	TGT CGT CAG CTC GTG	Sequencing	[21]
	1050R	CAC GAG CTG ACG ACA	Sequencing	[21]
18S rRNA	NS5 (F)	AAC TTA AAG GAA TTG ACG GAA G	PCR and Sequencing	[17]
	NS6 (R)	GCA TCA CAG ACC TGT TAT TGC CTC	PCR and Sequencing	[17]
*Corynebacteria rpoB*	C2700F	CGW ATG AAC ATY GGB CAG GT	PCR and Sequencing	[18]
	C1330R	TCC ATY TCR CCR AAR CGC TG	PCR and Sequencing	[18]
*Enterobacteria rpoB*	CM7 (F)	AAC CAG TTC CGC GTT GGC CTG G	PCR and Sequencing	[19]
	CM31b (R)	CCT GAA CAA CAC GCT CGG A	PCR and Sequencing	[19]
*Staphylococci rpoB*	Staph_F	AAC CAA TTC CGT ATI GGT TT	PCR and Sequencing	[20]
	Staph_R	CCG TCC CAA GTC ATG AAA C	PCR and Sequencing	[20]
*Streptococci rpoB*	Strepto_F	AAR YTI GGM CCT GAA GAA AT	PCR and Sequencing	[14]
	Strepto_R	TGI ART TTR TCA TCA AAC ATG TG	PCR and Sequencing	[14]

## Results

Overall, 191 out of 202 clinical isolates from Senegal were included in the analyses; 11 isolates were excluded from the analysis due to either contamination or an inability to be cultured in France. False-positive identification at the species level was observed for 6.3% (11/175) of identified isolates in Dakar and for 4.8% (9/184) in Marseille. No false-positive identification was observed at the genus level in Marseille and Dakar. Data details of microbial identification are shown in S1 Table.

### Concordant identification between the 2 MALDI-TOF MSs at the species level

Overall, 153 out of 191 isolates (80.1%) yielded an accurate and concordant identification at the species level with the 2 different MALDI-TOF MSs in Dakar and Marseille. The 153 isolates correctly identified at the species level by the two MALDI-TOF MSs were from 4 phyla (Table 2), with 63 *Firmicutes* (40.8%), 83 *Proteobacteria* (54.6%), 3 *Actinobacteria* (2%), and 4 *fungi* (2.6%). Among the 63 *Firmicutes*, 1 *Aerococcus viridans*; 2 *Bacillus megaterium*; 7 isolates of *Enterococcus*, including *Enterococcus faecalis* and *Enterococcus faecium*; 36 *Staphylococcus*; and 16 isolates of *Streptococcus*, including 11 *Streptococcus agalactiae*, were properly identified.

**Table 2:** 153 clinical isolates correctly identified at the species level by MALDI-TOF MS, first in Dakar (Senegal) and then in Marseille (France).

Phylum	Genus	Species	No. of isolates
*Actinobacteria*	*Corynebacterium*	*Corynebacterium amycolatum*	2
		*Corynebacterium striatum*	1
*Firmicutes*	*Aerococcus*	*Aerococcus viridans*	1
	*Bacillus*	*Bacillus megaterium*	2
	*Enterococcus*	*Enterococcus faecalis*	6
		*Enterococcus faecium*	1
	*Staphylococcus*	*Staphylococcus arlettae*	1
		*Staphylococcus aureus*	9
		*Staphylococcus capitis*	1
		*Staphylococcus caprae*	1
		*Staphylococcus cohnii*	4
		*Staphylococcus epidermidis*	1
		*Staphylococcus haemolyticus*	9
		*Staphylococcus hominis*	4
		*Staphylococcus saprophyticus*	2
		*Staphylococcus simulans*	2
		*Staphylococcus warneri*	2
	*Streptococcus*	*Streptococcus agalactiae*	11
		*Streptococcus anginosus*	1
		*Streptococcus dysgalactiae*	1
		*Streptococcus pyogenes*	3
		*Streptococcus salivarius*	1
*Proteobacteria*	*Acinetobacter*	*Acinetobacter baumannii*	9
		*Acinetobacter lwoffii*	1
		*Acinetobacter radioresistens*	3
	*Citrobacter*	*Citrobacter koseri*	2
	*Enterobacter*	*Enterobacter cloacae*	14
		*Enterobacter gergoviae*	2
	*Escherichia*	*Escherichia coli*	10
	*Klebsiella*	*Klebsiella oxytoca*	2
		*Klebsiella pneumoniae*	10
	*Kluyvera*	*Kluyvera ascorbata*	1
	*Haemophilus*	*Haemophilus influenzae*	1
	*Morganella*	*Morganella morganii*	5
	*Proteus*	*Proteus mirabilis*	4
		*Proteus penneri/vulgaris*	2
	*Pseudomonas*	*Pseudomonas aeruginosa*	11
		*Pseudomonas putida*	1
	*Salmonella*	*Salmonella enterica*	3
	*Serratia*	*Serratia marcescens*	1
	*Stenotrophomonas*	*Stenotrophomonas maltophilia*	1
*Fungi*	*Candida*	*Candida albicans*	3
		*Candida tropicalis*	1
**Total**			**153**

Among the 83 *Proteobacteria*, 56 *Enterobacteria*, 26 non-fermentative Gram-negative bacteria, and 1 *Haemophilus influenzae* were correctly identified. Among the 56 *Enterobacteria*, 14 *Enterobacter cloacae*, 10 *Klebsiella pneumoniae*, 10 *Escherichia coli*, 5 *Morganella morganii*, 4 *Proteus mirabilis*, 3 *Salmonella enterica*, 2 *Citrobacter koseri*, 2 *Enterobacter georgoviae*, 2 *Klebsiella oxytoca*, 1 *Serratia marcescens*, and 1 *Kluyvera ascorbata* were correctly identified. Among the 26 non-fermentative Gram-negative bacteria, 11 *Pseudomonas aeruginosa* and 9 *Acinetobacter baumannii* were properly identified. Among the 3 *Actinobacteria*, 2 *Corynebacterium amycolatum* and 1 *Corynebacterium striatum* were correctly identified. Four fungi, including 3 *Candida albicans* and 1 *Candida tropicalis*, were also properly identified.

### Concordant identification between the 2 MALDI-TOF MSs at only the genus level

Overall, 174 out of 191 isolates (91.1%) yielded an accurate and concordant identification at the genus level. Among them, 21 isolates were only concordant at the genus level but not at the species level (Table 3). One isolate of oral streptococci (*Streptococcus oralis*) was not properly identified with either of the MALDI-TOF MSs.

**Table 3:** Twenty-one MALDI-TOF MS identifications with concordance only at the genus level in Dakar (Senegal) and Marseille (France); Species identification was resolved by molecular biology analyses coupled with phenotypic tests for the *Bacillus cereus* group. No: number of isolates, if greater than 1

MALDI-TOF MS, Dakar (No.)	MALDI-TOF MS, Marseille (No.)	Identification using molecular biology tools
*Achromobacter denitrificans* (5)	*Achromobacter xylosoxidans* (5)	*A*. *xylosoxidans* ^1^ (5) [HQ288926: 98.8% and 99.3%; GQ889256: 99.6%; AF411020: 100% and 99.9%]*
*Acinetobacter baumannii*	*Acinetobacter junii*	*A*. *junii* ^1^ [HE651919: 99%]
*Corynebacterium amycolatum*	*Corynebacterium striatum*	*C*. *striatum* ^2^ [HE586297: 99.7%]
*Staphylococcus aureus*	*Staphylococcus haemolyticus*	*S*. *haemolyticus* ^1^ [NR_074994: 99.3%]
*Staphylococcus haemolyticus*	*Staphylococcus simulans*	*S*. *simulans* ^1^ [KC849411: 97.9%]
*Streptococcus anginosus*	*Streptococcus constellatus*	*S*. *constellatus* ^4^ [AF535184: 98.2%]
*Acinetobacter* sp.	*A*. *baumannii*	*A*. *baumannii* ^1^ [EU734813: 97.8%]
*Enterobacter* sp. (3)	*Enterobacter cloacae* (3)	*E*. *cloacae* ^3^ (3) [JQ435865: 97.9%; 99.1% and 98.1%]
*Burkholderia* sp.	*Burkholderia cepacia*	*B*. *cepacia* ^1^ [EU742139: 99.2%]
*Streptococcus gordonii*	*Streptococcus mitis*	*Streptococcus oralis* ^4^ [AF535168: 98%]
*Bacillus cereus*	*Bacillus anthracis*	*B*. *cereus* group^1^ ^,^ ^§^ [CP003187: 97.9%]
*Bacillus cereus*	*Bacillus* sp.	*B*. *cereus* group^1^ ^,^ ^§^ [CP003187: 99.3%]
*Enterobacter georgoviae*	*Enterobacter asburiae*	*E*. *georgoviae* ^3^ [AJ566945: 99%]
*Salmonella enterica*	*Salmonella Typhi*	*S*. *enterica* serovar Paratyphi A^3^ [JQ728878: 100%]
*Staphylococcus warneri*	*Staphylococcus pasteuri*	*S*. *warneri* ^1^ [HQ407248: 99%]

^1^ 16S rRNA sequencing

^2^ *rpoB* gene sequencing for *Corynebacteria*

^3^ *rpoB* gene sequencing for *Enterobacteria*

^4^ *rpoB* gene sequencing for *Streptococci*

* The GenBank accession numbers and the percentage of homology obtained for all the sequenced bacteria are given between the brackets.

^§^ The isolates were both hemolytic on Columbia blood agar, and no parasporal crystals were observed in sporulated cultures, leading to the identification of *Bacillus cereus*.

Five isolates were correctly identified with the MALDI-TOF MS in Dakar but not with the MS in Marseille: 2 isolates of *Bacillus cereus*, 1 *Enterobacter gergoviae*, 1 *Salmonella* subsp. *enterica* serovar Paratyphi A, and 1 *Staphyloccus warneri*.

Fifteen isolates were properly identified with the MALDI-TOF MS in Marseille but not with the MS in Dakar. Among these, 5 isolates of *Achromobacter xylosoxidans* were systematically inaccurately identified as *Achromobacter denitrificans*. Three isolates of *E*. *cloacae* was only identified as *Enterobacter* sp. One isolate of *Burkholderia cepacia* and 1 isolate of *Acinetobacter baumannii* were also only identified at the genus level. One isolate of *Corynebacterium striatum* was incorrectly identified as *C*. *amycolatum*, and 1 isolate of *Streptococcus constellatus* was misidentified as *S*. *anginosus*. Finally, 1 isolate of *S*. *haemolyticus* and 1 isolate of S. *simulans* were also inaccurately identified (Table 3).

### Lack of identification by the MALDI-TOF MS in Dakar or in Marseille

**Fig 1:**
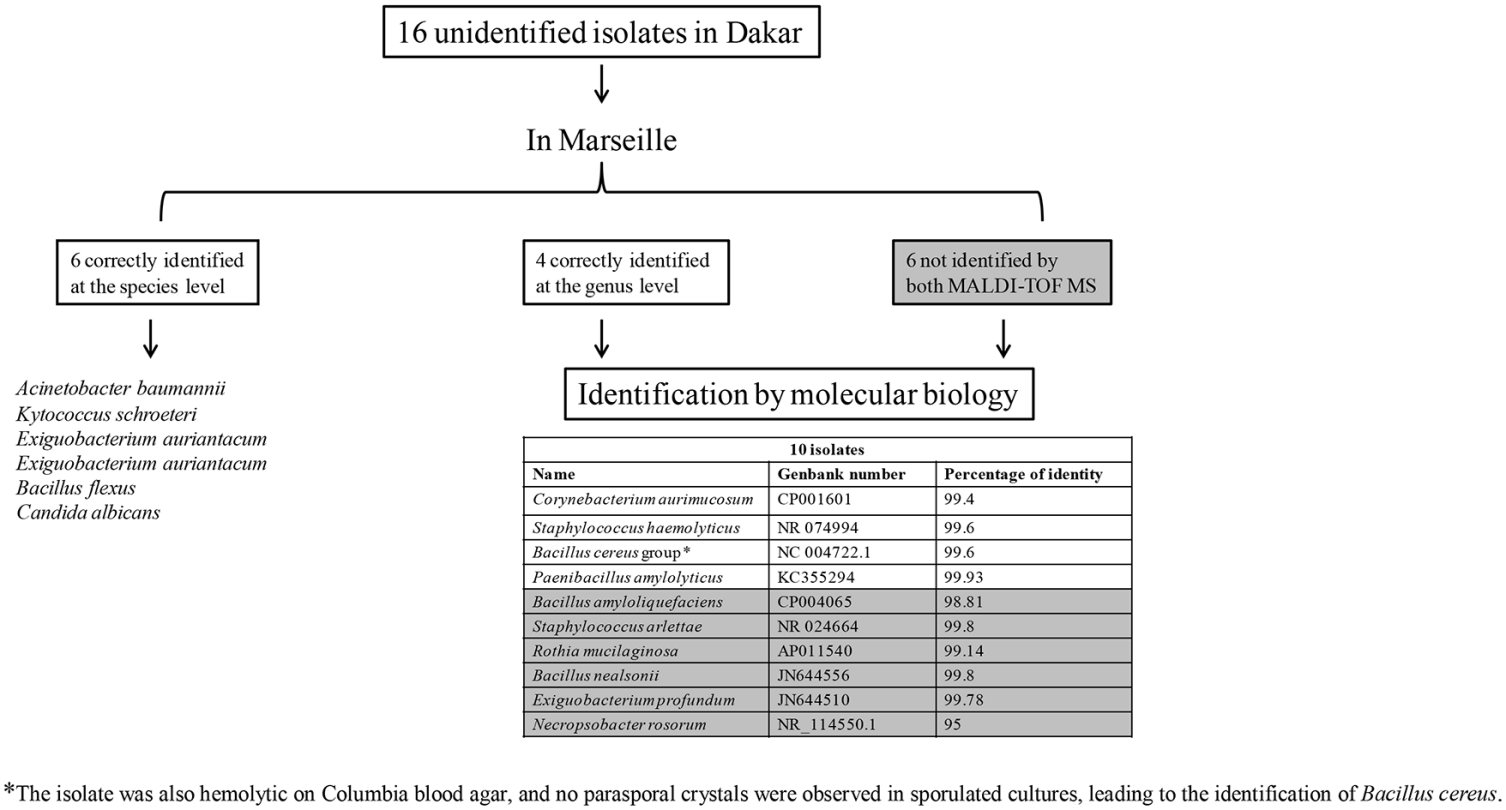
Sixteen clinical isolates not identified in Dakar (Senegal).

One isolate was accurately identified as *Escherichia hermannii* in Dakar but not in Marseille; PCR and sequencing of the *rpoB* sequence confirmed the identification of *Escherichia hermannii*. Sixteen out of 191 (8.4%) isolates were not identified by MALDI-TOF MS in Dakar (Fig 1). Among these 16 isolates, 6 were correctly identified at the species level in Marseille (2 isolates of *Exiguobacterium aurantiacum*, 1 isolate of *A*. *baumannii*, 1 of *Kytococcus schroeteri*, 1 of *Bacillus flexus*, and 1 of *C*. *albicans*). Four isolates were only identified at the genus level in Marseille, and molecular biology with sequencing was necessary to allow their identification (1 isolate of *Corynebacterium aurimucosum*, 1 of *S*. *haemolyticus*, 1 of *Paenibacillus amylolyticus*, and 1 of *B*. *cereus*). Finally, 6 (3%) isolates were not identified with either MALDI-TOF MS. PCR followed by sequencing allowed the identification of 1 isolate of *Rothia mucilaginosa*, 1 of *Staphylococcus arlettae*, 1 of *Bacillus amyloliquefaciens*, 1 of *Bacillus nealsonii*, and 1 of *Exiguobacterium profundum*. In addition, the last isolate exhibited 95% 16S rRNA nucleotide sequence identity with *Necropsobacter rosorum* (NR_114550.1), the phylogenetically closest validated species (Fig 1), suggesting that this isolate corresponded to a new bacterial species, which we named “*Necropsobacter massiliensis*”. The Genbank accession number for the 16S rRNA sequence of “*Necropsobacter massiliensis*” is HG428679. The full genome sequencing and the characterization of “*Necropsobacter massiliensis*” have been performed [25].

## Discussion

MALDI-TOF MS has become a revolutionary technique in the world of clinical microbiology, allowing the quick and accurate identification of clinical pathogenic microorganisms, including bacteria and fungi [3,5,26]. Thus, this newly developed diagnostic tool is increasingly being employed in clinical microbiology laboratories. Because microbe identification is sometimes difficult in Africa, we have tested a MALDI-TOF MS in Dakar, Senegal. The impact of the installation of this MALDI-TOF MS was so great that other phenotypic-based identification was immediately stopped. Data obtained in this study have confirmed that MALDI-TOF MS is an accurate tool for microbial identification to hospital in Dakar. Indeed, in total, 80.1% and 91% of isolates were accurately identified at the species and genus levels, respectively, using MALDI-TOF MS in Dakar. These data correspond well to previous work performed in our laboratory in Marseille, France (84.1% of 1,660 tested isolates accurately identified at the species level) [5].

*E*. *coli*, *K*. *pneumoniae*, *S*. *agalactiae*, *A*. *baumannii*, *P*. *aeruginosa*, *S*. *aureus*, *S*. *haemolyticus*, *E*. *cloacae*, *E*. *faecalis*, and *S*. *epidermidis* are the 10 most commonly identified bacteria in the laboratory of the Hôpital Principal de Dakar (personal data from the hospital), representing 94.2% of all bacteria routinely identified in the laboratory. All these bacteria were accurately identified with the MALDI-TOF MS in Dakar. Thus, MALDI-TOF MS is a powerful tool for routine bacterial identification in Africa because it allows for the rapid identification of the species most frequently observed there.

The most frequent misidentification observed in Dakar was at the species level for *Achromobacter xylosoxidans*, which was systematically misidentified as *Achromobacter denitrificans* with the Saramis software [27]. Rare misidentifications of other non-fermentative Gram-negative bacteria, such as *Acinetobacter* spp. and *Burkholderia* spp., were also observed. Overall, these misidentifications were comparable to those in other studies that evaluated MALDI-TOF MS performance for the identification of non-fermentative Gram-negative bacteria [27]. Among the *Enterobacteria*, 1 isolate of *Salmonella enterica* serovar *Paratyphi* was misidentified in Marseille as *Salmonella enterica Typhi;* this is probably linked to the fact that these species are so similar at the proteomic level that their MALDI-TOF spectra are sometimes hardly distinguishable. Besides, 3 isolates of *E*. *cloacae* were identified only at the genus level in Dakar. These data are surprising because 16 other isolates of *E*. *cloacae* were properly identified. This discrepancy may be explained by the fact that all these 3 isolates produced extended-spectrum beta-lactamases. Indeed, it seems that the Saramis database version 4.0 sometimes has difficulties identifying extended-spectrum beta-lactamase-producing *Enterobacter* at the species level; this was not reported using the Vitek MS *in vitro* diagnostic (IVD) system with the IVD database version 2.0 and MYLA software [28]. Poor identifications are also sometimes associated with technically poor preparation linked to the quality of deposits and impurities of the deposited colony [2]. New deposits might allow an accurate identification.

Coryneform bacteria include a diverse range of bacterial genera grouped together as aerobic non-spore-forming Gram-positive bacilli. Of these, the genus *Exiguobacterium* was first isolated from potato-processing effluent in 1983 and identified as *Exiguobacterium aurantiacum* [29]. *E*. *aurantiacum* has only been recently reported as a human pathogen with six isolates obtained from patients with bacteremia [29]. Its lack of identification in Dakar was due to its absence from the database. Another bacterium from the genus *Exiguobacterium*, *Exiguobacterium profundum*, was not identified by either MALDI-TOF. *Exiguobacterium profundum* is a recently discovered bacterial species that was first described in 2007 [30]. It was first reported in humans in 2013 with an isolate obtained from a patient with bacteremia and identified using 16S rRNA sequencing [31]. *Exiguobacterium profundum* was also absent from the database.

Several misidentifications or non-identifications of *Bacillus* species were also observed in Marseille and Dakar. The main limit was observed among the *Bacillus cereus* group [32,33]. These identification difficulties may be linked to the fact that these bacteria have very similar spectral profiles associated with the presence of common protein biomarkers [32]. In addition, on the basis of genetic evidence, it has been proposed that *B*. *anthracis*, *B*. *cereus*, and *B*. *thuringiensis* belong to the same species (*B*. *cereus* sensu lato), but the status of separate species has been retained due to their remarkably different virulence [34]. Neither MALDI-TOF MS was able to identify *Bacillus nealsonii*, and *Bacillus flexus* was only identified in Marseille. These identification problems were linked to the absence of spectra for these bacteria in the databases. One isolate of *Staphyloccus warneri* was misidentified as *Staphyloccus pasteuri* in Marseille. As *S*. *pasteuri* is phenotypically the closest species to *S*. *warneri*, close MS spectra between the 2 species has lead probably to a misidentification. In addition, both MALDI-TOF MSs failed twice to identify oral *Streptococci* accurately at the species level; these difficulties have been reported previously [1,3,5,6,35]. Finally, on the basis of the data observed in Dakar during this study (showing for example that *A*. *xylosoxidans* has been systematically falsely identified as *A*. *denitrificans*) and those of the literature which help to better interpret the data, we speculate that no more than 3% of false-positive species identifications might be detected in the real-life application of MALDI-TOS MS in Dakar [9].

The improvement of microbial identification is based on the regular addition of new spectra to the databases. For example, in our study, an isolate of *K*. *schroeteri* was not identified in Dakar, whereas it was identified in Marseille. In fact, an isolate of *K*. *schroeteri* was first cultivated from a valvular biopsy of a patient with endocarditis in our hospital [36]. This isolate was identified using PCR and 16S rRNA sequencing [36], and its spectrum was added to our database. Subsequently, other isolates of *K*. *schroeteri* were obtained in a human gut microbiome study performed by our team, and their spectra have been systematically added in our database [37]. Overall, MALDI-TOF MS databases are expanding at a rapid rate, allowing the improvement of the identification but also limiting the value of comparisons of the current results with those of older studies. Finally, 1 of the isolates unidentified by the 2 MALDI-TOF MSs corresponds to a new species of the genus *Necropsobacter* and the *Pasteurellaceae* family. It was named *Necropsobacter massiliensis*, and its full genome sequence is currently being annotated [25].

Of note, MALDI-TOF MS proposes sometimes more than one confident species identification. Indeed, certain species are so similar at the proteomic level that their spectra are sometimes hardly distinguishable. For example, for a same colony, several confident identifications such as *E*. *cloacae*, *E*. *asburiae*, and *E*. *kobei* can be observed. In this case, *E*. *cloacae* complex should be the identification. A similar situation can be observed with *Streptococcus pneumoniae* and other bacteria of the viridans group of streptococci requiring the realization of *S*. *pneumoniae* latex agglutination and/or optochin susceptibility. Another similar pitfall of MS is reported for some strains of *E*. *coli* and *Shigella*; complementary tests including lactose fermentation and quick indole are thus required. Finally, we do not yet know if the discrimination of some so closely related microorganisms could be performed with the improvement of databases and/or software [10].

Currently, 2 different MALDI-TOF MS are widely commercialized. The mass spectrometer of bioMérieux is a large floor model whereas that of Bruker is a desktop instrument. Each system includes spot target plates but the plates are reusable steel targets for Bruker whereas those of bioMérieux are disposable plastic targets. More recently, an extensive comparison was performed on the 2 available MALDI-TOF MS platforms in USA with the most current database versions (Vitek MS and Bruker Microflex LT MALDI-TOF MS). The 2 systems were analytically comparable, with overall isolate identification well above 90% for Food and Drug Administration (FDA)-approved and non-FDA-approved species [9,10]. Both instruments received high ratings in the user evaluation. However, 75% of the evaluating technologists favored the Viteck MS in a qualitative user assessment.

The robust microbe identification by MALDI-TOF MS in both Dakar and Marseille demonstrates that MALDI-TOF MS can be used as a first-line tool in clinical microbiology laboratories world-wide.

## Supporting Information

S1 Table Data of microbial identification using MALDI-TOF MS in Dakar and Marseille plus molecular biology in Marseille. (PDF)
