# Supplementary material for: MALDI-TOF Mass Spectrometry: A Powerful Tool for Clinical Microbiology at Hôpital Principal de Dakar, Senegal (West Africa)
Source: PLoS One. 2015 Dec 30;10(12):e0145889. doi: 10.1371/journal.pone.0145889 (PMC4696746; doi:10.1371/journal.pone.0145889)
Supplement: S1 Table — (PDF) [file pone.0145889.s001.pdf]

MALDI-TOF Dakar		MALDI-TOF Marseille		Molecular biology Marseille	Final identification
**Identified strains**	**Score**	**Identified strains**	**Score**	**Identified strains with closest Blast in GenBank**	
Achromobacter denitrificans	81,2	*Achromobacter xylosoxidans*	2.213	*Achromobacter xylosoxidans*[HQ288926 (99.3%)]	*Achromobacter xylosoxidans*
Achromobacter denitrificans	90,5	*Achromobacter xylosoxidans*	2.014	*Achromobacter xylosoxidans* [HQ288926 (98.8%)]	*Achromobacter xylosoxidans*
Achromobacter denitrificans	89,9	*Achromobacter xylosoxidans*	2.059	*Achromobacter xylosoxidans* [GQ889256 (99.6%)]	*Achromobacter xylosoxidans*
Achromobacter denitrificans	75,1	*Achromobacter xylosoxidans*	2.105	*Achromobacter xylosoxidans* [AF411020 (100%)]	*Achromobacter xylosoxidans*
Achromobacter denitrificans	77,4	*Achromobacter xylosoxidans*	2.093	*Achromobacter xylosoxidans*[AF411020 (99.9%)]	*Achromobacter xylosoxidans*
Acinetobacter baumannii	83,6	*Acinetobacter baumannii*	2.422		*Acinetobacter baumannii*
Acinetobacter baumannii	93,1	*Acinetobacter baumannii*	2.391		*Acinetobacter baumannii*
Acinetobacter baumannii	93,3	*Acinetobacter baumannii*	2.223		*Acinetobacter baumannii*
Acinetobacter baumannii	96,5	*Acinetobacter baumannii*	2.414		*Acinetobacter baumannii*
Acinetobacter baumannii	96,6	*Acinetobacter baumannii*	2.338		*Acinetobacter baumannii*
Acinetobacter baumannii	78	*Acinetobacter baumannii*	2.255		*Acinetobacter baumannii*
Acinetobacter baumannii	90,3	*Acinetobacter baumannii*	2.416		*Acinetobacter baumannii*
Acinetobacter baumannii	84,8	*Acinetobacter baumannii*	2.253		*Acinetobacter baumannii*
Acinetobacter baumannii	78,3	*Acinetobacter baumannii*	2.213		*Acinetobacter baumannii*
Acinetobacter baumannii	80	*Acinetobacter junii*	2.132	*Acinetobacter junii* [HE651919 (99%)]	*Acinetobacter junii*
Acinetobacter lwoffii	99,9	*Acinetobacter lwoffii*	2.205		*Acinetobacter lwoffii*
Acinetobacter radioresistens	87,6	*Acinetobacter radioresistens*	2.233		*Acinetobacter radioresistens*
Acinetobacter radioresistens	99,9	*Acinetobacter radioresistens*	2.164		*Acinetobacter radioresistens*
Acinetobacter radioresistens	94	*Acinetobacter radioresistens*	2.176		*Acinetobacter radioresistens*
*Acinetobacter*sp	82	*Acinetobacter baumannii*	2.303	*Acinetobacter baumannii* [EU734813 (97.8%)]	*Acinetobacter baumannii*
Aerococcus viridans	96,1	*Aerococcus viridans*	2.241		*Aerococcus viridans*
Bacillus cereus	96,9	*Bacillus anthracis*	1.987	*Bacillus thuringiensis* [CP003187 (97.9%)]	*Bacillus thuringiensis*
Bacillus cereus	99,9	*Bacillus* sp	2.25	*Bacillus cereus* [CP003187 (99.3%)]	*Bacillus cereus*
Bacillus megaterium	92,4	*Bacillus megaterium*	2.212		*Bacillus megaterium*
Bacillus megaterium	97,8	*Bacillus megaterium*	2.031		*Bacillus megaterium*
Burkholderia sp	83,2	*Burkholderia cepacia*	2.271	*Burkholderia cepacia* [EU742139 (99.2%)]	*Burkholderia cepacia*

Candida albicans	99,9	*Candida albicans*	1.904		*Candida albicans*
Candida albicans	99,9	*Candida albicans*	1.989		*Candida albicans*
Candida albicans	95,6	*Candida albicans*	1.921		*Candida albicans*
Candida tropicalis	99,2	*Candida tropicalis*	1.936		*Candida tropicalis*
Citrobacter koseri	79,2	*Citrobacter koseri*	2.408		*Citrobacter koseri*
Citrobacter koseri	95,7	*Citrobacter koseri*	2.534		*Citrobacter koseri*
Corynebacterium amycolatum	99,5	*Corynebacterium amycolatum*	2.191		*Corynebacterium amycolatum*
Corynebacterium amycolatum	99,9	*Corynebacterium amycolatum*	2.023		*Corynebacterium amycolatum*
Corynebacterium amycolatum	99,9	*Corynebacterium striatum*	2.052	*Corynebacterium striatum* [HE586297 (99.7%)]	*Corynebacterium striatum*
Corynebacterium striatum	99,9	*Corynebacterium striatum*	2.136		*Corynebacterium striatum*
Enterobacter cloacae	76,8	*Enterobacter cloacae*	2.136		*Enterobacter cloacae*
Enterobacter cloacae	85,2	*Enterobacter cloacae*	2.137		*Enterobacter cloacae*
Enterobacter cloacae	88,6	*Enterobacter cloacae*	2.068		*Enterobacter cloacae*
Enterobacter cloacae	78	*Enterobacter cloacae*	2.121		*Enterobacter cloacae*
Enterobacter cloacae	87	*Enterobacter cloacae*	2.093		*Enterobacter cloacae*
Enterobacter cloacae	81,8	*Enterobacter cloacae*	2.268		*Enterobacter cloacae*
Enterobacter cloacae	91,8	*Enterobacter cloacae*	2.243		*Enterobacter cloacae*
Enterobacter cloacae	85,2	*Enterobacter cloacae*	2.298		*Enterobacter cloacae*
Enterobacter cloacae	92,1	*Enterobacter cloacae*	2.313		*Enterobacter cloacae*
Enterobacter cloacae	81,9	*Enterobacter cloacae*	2.243		*Enterobacter cloacae*
Enterobacter cloacae	99,9	*Enterobacter cloacae*	2.177		*Enterobacter cloacae*
Enterobacter cloacae	87,7	*Enterobacter cloacae*	2.328		*Enterobacter cloacae*
Enterobacter cloacae	80,3	*Enterobacter cloacae*	2.178		*Enterobacter cloacae*
Enterobacter cloacae	81	*Enterobacter cloacae*	2.274		*Enterobacter cloacae*
Enterobacter georgoviae	93	*Enterobacter georgoviae*	2.126		*Enterobacter gergoviae*
Enterobacter georgoviae	96,8	*Enterobacter georgoviae*	2.151		*Enterobacter gergoviae*
Enterobacter georgoviae	99	*Enterobacter asburiae*	2.121	*Enterobacter georgoviae* [AJ566945 (99%)]	*Enterobacter georgoviae*
Enterobacter sp	91,8	*Enterobacter cloacae*	2.196	*Enterobacter cloacae* [JQ435865 (97.9%)]	*Enterobacter cloacae*

Enterobacter sp	87,9	*Enterobacter cloacae*	2.031	*Enterobacter cloacae* [JQ435865 (99.1%)]	*Enterobacter cloacae*
Enterobacter sp	84	*Enterobacter cloacae*	2.177	*Enterobacter cloacae* [JQ435865 (98.1%)]	*Enterobacter cloacae*
Enterococcus faecalis	99,9	*Enterococcus faecalis*	2.375		*Enterococcus faecalis*
Enterococcus faecalis	99,9	*Enterococcus faecalis*	2.231		*Enterococcus faecalis*
Enterococcus faecalis	99,9	*Enterococcus faecalis*	2.373		*Enterococcus faecalis*
Enterococcus faecalis	99,9	*Enterococcus faecalis*	2.136		*Enterococcus faecalis*
Enterococcus faecalis	90,3	*Enterococcus faecalis*	2.376		*Enterococcus faecalis*
Enterococcus faecalis	99,9	*Enterococcus faecalis*	2.449		*Enterococcus faecalis*
Enterococcus faecium	99,2	*Enterococcus faecium*	2.423		*Enterococcus faecium*
Escherichia coli	99,9	*Escherichia coli*	2.227		*Escherichia coli*
Escherichia coli	92,4	*Escherichia coli*	2.198		*Escherichia coli*
Escherichia coli	99,9	*Escherichia coli*	2.306		*Escherichia coli*
Escherichia coli	93,8	*Escherichia coli*	2.295		*Escherichia coli*
Escherichia coli	87,5	*Escherichia coli*	2.153		*Escherichia coli*
Escherichia coli	95,4	*Escherichia coli*	2.271		*Escherichia coli*
Escherichia coli	97,8	*Escherichia coli*	2.277		*Escherichia coli*
Escherichia coli	99,9	*Escherichia coli*	2.228		*Escherichia coli*
Escherichia coli	88,1	*Escherichia coli*	2.261		*Escherichia coli*
Escherichia coli	95,4	*Escherichia coli*	2.228		*Escherichia coli*
Escherichia hermannii	97,1	No reliable identification	No score	*Escherichia hermannii* [JN175345 (98,82%)]	*Escherichia hermannii*
Haemophilus influenzae	96,5	*Haemophilus influenzae*	2.274		*Haemophilus influenzae*
Klebsiella oxytoca	97,1	*Klebsiella oxytoca*	2.202		*Klebsiella oxytoca*
Klebsiella oxytoca	93,8	*Klebsiella oxytoca*	2.199		*Klebsiella oxytoca*
Klebsiella pneumoniae	96,4	*Klebsiella pneumoniae*	2.409		*Klebsiella pneumoniae*
Klebsiella pneumoniae	93,8	*Klebsiella pneumoniae*	2.298		*Klebsiella pneumoniae*
Klebsiella pneumoniae	99	*Klebsiella pneumoniae*	2.283		*Klebsiella pneumoniae*
Klebsiella pneumoniae	83,3	*Klebsiella pneumoniae*	2.366		*Klebsiella pneumoniae*
Klebsiella pneumoniae	86	*Klebsiella pneumoniae*	2.348		*Klebsiella pneumoniae*

Klebsiella pneumoniae	91,2	*Klebsiella pneumoniae*	2.444		*Klebsiella pneumoniae*
Klebsiella pneumoniae	76,4	*Klebsiella pneumoniae*	2.391		*Klebsiella pneumoniae*
Klebsiella pneumoniae	99,9	*Klebsiella pneumoniae*	2.126		*Klebsiella pneumoniae*
Klebsiella pneumoniae	82,7	*Klebsiella pneumoniae*	2.159		*Klebsiella pneumoniae*
Klebsiella pneumoniae	83,4	*Klebsiella pneumoniae*	2.238		*Klebsiella pneumoniae*
Kluyvera ascorbata	99,8	*Kluyvera ascorbata*	2.123		*Kluyvera ascorbata*
Morganella morganii	96,4	*Morganella morganii*	2.452		*Morganella morganii*
Morganella morganii	99,9	*Morganella morganii*	2.549		*Morganella morganii*
Morganella morganii	92,6	*Morganella morganii*	2.526		*Morganella morganii*
Morganella morganii	98	*Morganella morganii*	2.632		*Morganella morganii*
Morganella morganii	92,4	*Morganella morganii*	2.511		*Morganella morganii*
No reliable identification	No score	*Acinetobacter baumannii*	2.391		*Acinetobacter baumannii*
No reliable identification	No score	*Kytococcus schroeteri*	2.202		*Kytococcus schroeteri*
No reliable identification	No score	*Exiguobacterium aurantiacum*	2.224		*Exiguobacterium aurantiacum*
No reliable identification	No score	*Exiguobacterium aurantiacum*	2.054		*Exiguobacterium aurantiacum*
No reliable identification	No score	*Bacillus flexus*	2.131		*Bacillus flexus*
No reliable identification	No score	*Candida albicans*	2.116		*Candida albicans*
No reliable identification	No score	*Corynebacterium aurimucosum*	1.874	*Corynebacterium aurimucosum* [CP001601 (99.4%)]	*Corynebacterium aurimucosum*
No reliable identification	No score	*Staphylococcus haemolyticus*	2.017	*Staphylococcus haemolyticus* [NR 074994 (99.6%)]	*Staphylococcus haemolyticus*
No reliable identification	No score	*Bacillus anthracis*	2.21	*Bacillus cereus* [CP003187 (100%)]	*Bacillus cereus*
No reliable identification	No score	*Paenibacillus barcinonensis*	1.9	*Paenibacillus amylolyticus* [KC355294 (99.93%)]	*Paenibacillus amylolyticus*
No reliable identification	No score	No reliable identification	No score	*Bacillus amyloliquefaciens* [CP004065 (98.81%)]	*Bacillus amyloliquefaciens*
No reliable identification	No score	No reliable identification	No score	*Staphylococcus arlettae* [NR 024664 (99.8%)]	*Staphylococcus arlettae*

No reliable identification	No score	No reliable identification	No score	*Rothia mucilaginosa* [AP011540 (99.14%)]	*Rothia mucilaginosa*
No reliable identification	No score	No reliable identification	No score	*Bacillus nealsonii* [JN644556 (99.80%)]	*Bacillus nealsonii*
No reliable identification	No score	No reliable identification	No score	*Exiguobacterium profundum* [JN644510 (99.78%)]	*Exiguobacterium profundum*
No reliable identification	No score	No reliable identification	No score	*Necropsobacter rosorum* [NR_114550.1 (94.8%)]	*Necropsobacter rosorum*
Proteus mirabilis	99,9	*Proteus mirabilis*	2.581		*Proteus mirabilis*
Proteus mirabilis	99,9	*Proteus mirabilis*	2.568		*Proteus mirabilis*
Proteus mirabilis	99,5	*Proteus mirabilis*	2.371		*Proteus mirabilis*
Proteus mirabilis	99,9	*Proteus mirabilis*	2.514		*Proteus mirabilis*
Proteus penneri/vulgaris	89,1	*Proteus penneri/vulgaris*	2.262		*Proteus penneri*
Proteus penneri/vulgaris	96,2	*Proteus penneri/vulgaris*	2.198		*Proteus vulgaris*
Pseudomonas aeruginosa	99,9	*Pseudomonas aeruginosa*	2.424		*Pseudomonas aeruginosa*
Pseudomonas aeruginosa	99,9	*Pseudomonas aeruginosa*	2.083		*Pseudomonas aeruginosa*
Pseudomonas aeruginosa	99,9	*Pseudomonas aeruginosa*	2.287		*Pseudomonas aeruginosa*
Pseudomonas aeruginosa	99,9	*Pseudomonas aeruginosa*	2.317		*Pseudomonas aeruginosa*
Pseudomonas aeruginosa	89,7	*Pseudomonas aeruginosa*	2.288		*Pseudomonas aeruginosa*
Pseudomonas aeruginosa	99,9	*Pseudomonas aeruginosa*	2.341		*Pseudomonas aeruginosa*
Pseudomonas aeruginosa	92	*Pseudomonas aeruginosa*	2.252		*Pseudomonas aeruginosa*
Pseudomonas aeruginosa	96	*Pseudomonas aeruginosa*	2.262		*Pseudomonas aeruginosa*
Pseudomonas aeruginosa	99,9	*Pseudomonas aeruginosa*	2.126		*Pseudomonas aeruginosa*
Pseudomonas aeruginosa	92	*Pseudomonas aeruginosa*	2.261		*Pseudomonas aeruginosa*
Pseudomonas aeruginosa	99,9	*Pseudomonas aeruginosa*	2.275		*Pseudomonas aeruginosa*
Pseudomonas putida	99,9	*Pseudomonas putida*	2.122		*Pseudomonas putida*
Salmonella enterica	99,9	*Salmonella paratyphi*	2.201		*Salmonella enterica subsp. enterica serovar Paratyphi A*
Salmonella enterica	99,9	*Salmonella paratyphi*	2.26		*Salmonella enterica subsp. enterica serovar Paratyphi A*
Salmonella enterica	84	*Salmonella paratyphi*	2.296		*Salmonella enterica subsp. enterica serovar Paratyphi A*

Salmonella enterica	78,3	*Salmonella typhi*	2.144	*Salmonella enterica* subsp. *enterica* serovar Paratyphi A [JQ728878 (100%)]	*Salmonella enterica subsp. enterica serovar Paratyphi A*
Serratia marcescens	99,9	*Serratia marcescens*	1.959		*Serratia marcescens*
Staphylococcus arlettae	96,1	*Staphylococcus arlettae*	2.224		*Staphylococcus arlettae*
Staphylococcus aureus	99,9	*Staphylococcus aureus*	2.345		*Staphylococcus aureus*
Staphylococcus aureus	99,9	*Staphylococcus aureus*	2.253		*Staphylococcus aureus*
Staphylococcus aureus	99,9	*Staphylococcus aureus*	2.275		*Staphylococcus aureus*
Staphylococcus aureus	99,9	*Staphylococcus aureus*	2.322		*Staphylococcus aureus*
Staphylococcus aureus	93,1	*Staphylococcus aureus*	2.398		*Staphylococcus aureus*
Staphylococcus aureus	99,9	*Staphylococcus aureus*	2.308		*Staphylococcus aureus*
Staphylococcus aureus	99,9	*Staphylococcus aureus*	2.257		*Staphylococcus aureus*
Staphylococcus aureus	99,9	*Staphylococcus aureus*	2.155		*Staphylococcus aureus*
Staphylococcus aureus	99,9	*Staphylococcus aureus*	2.003		*Staphylococcus aureus*
Staphylococcus aureus	94,9	*Staphylococcus haemolyticus*	2.117	*Staphylococcus haemolyticus* [NR_074994 (99.3%)]	*Staphylococcus haemolyticus*
Staphylococcus capitis	96,9	*Staphylococcus capitis*	1.927		*Staphylococcus capitis*
Staphylococcus caprae	99,9	*Staphylococcus caprae*	2.039		*Staphylococcus caprae*
Staphylococcus cohnii	96,5	*Staphylococcus cohnii*	1.929		*Staphylococcus cohnii*
Staphylococcus cohnii	84	*Staphylococcus cohnii*	2.034		*Staphylococcus cohnii*
Staphylococcus cohnii	95	*Staphylococcus cohnii*	2.111		*Staphylococcus cohnii*
Staphylococcus cohnii	99,9	*Staphylococcus cohnii*	2.034		*Staphylococcus cohnii*
Staphylococcus epidermidis	99,9	*Staphylococcus epidermidis*	2.065		*Staphylococcus epidermidis*
Staphylococcus haemolyticus	84	*Staphylococcus haemolyticus*	2.105		*Staphylococcus haemolyticus*
Staphylococcus haemolyticus	92,1	*Staphylococcus haemolyticus*	2.178		*Staphylococcus haemolyticus*
Staphylococcus haemolyticus	99,9	*Staphylococcus haemolyticus*	2.118		*Staphylococcus haemolyticus*
Staphylococcus haemolyticus	96,2	*Staphylococcus haemolyticus*	2.065		*Staphylococcus haemolyticus*
Staphylococcus haemolyticus	96,4	*Staphylococcus haemolyticus*	2.097		*Staphylococcus haemolyticus*
Staphylococcus haemolyticus	99,9	*Staphylococcus haemolyticus*	2.152		*Staphylococcus haemolyticus*
Staphylococcus haemolyticus	84,1	*Staphylococcus haemolyticus*	2.158		*Staphylococcus haemolyticus*
Staphylococcus haemolyticus	88,1	*Staphylococcus haemolyticus*	2.181		*Staphylococcus haemolyticus*

Staphylococcus haemolyticus	84,5	*Staphylococcus haemolyticus*	2.059		*Staphylococcus haemolyticus*
Staphylococcus haemolyticus	99,9	*Staphylococcus simulans*	2.273	*Staphylococcus simulans* [KC849411 (97.9%)]	*Staphylococcus simulans*
Staphylococcus hominis	99,9	*Staphylococcus hominis*	2.277		*Staphylococcus hominis*
Staphylococcus hominis	93	*Staphylococcus hominis*	2.121		*Staphylococcus hominis*
Staphylococcus hominis	93,6	*Staphylococcus hominis*	2.248		*Staphylococcus hominis*
Staphylococcus hominis	99,9	*Staphylococcus hominis*	2.199		*Staphylococcus hominis*
Staphylococcus saprophyticus	96,2	*Staphylococcus saprophyticus*	2.003		*Staphylococcus saprophyticus*
Staphylococcus saprophyticus	99,9	*Staphylococcus saprophyticus*	2.013		*Staphylococcus saprophyticus*
Staphylococcus simulans	99,9	*Staphylococcus simulans*	2.137		*Staphylococcus simulans*
Staphylococcus simulans	99,4	*Staphylococcus simulans*	2.171		*Staphylococcus simulans*
Staphylococcus warneri	99,9	*Staphylococcus warneri*	2.216		*Staphylococcus warneri*
Staphylococcus warneri	97,5	*Staphylococcus warneri*	2.187		*Staphylococcus warneri*
Staphylococcus warnerii	95	*Staphylococcus pasteuri*	2.041	*Staphylococcus warneri* [HQ407248 (99%)]	*Staphylococcus warneri*
Stenotrophomonas maltophilia	99,9	*Stenotrophomonas maltophilia*	2.094		*Stenotrophomonas maltophilia*
Streptococcus agalactiae	94,7	*Streptococcus agalactiae*	2.231		*Streptococcus agalactiae*
Streptococcus agalactiae	97,4	*Streptococcus agalactiae*	2.416		*Streptococcus agalactiae*
Streptococcus agalactiae	94,5	*Streptococcus agalactiae*	2.155		*Streptococcus agalactiae*
Streptococcus agalactiae	84,1	*Streptococcus agalactiae*	2.414		*Streptococcus agalactiae*
Streptococcus agalactiae	99,9	*Streptococcus agalactiae*	2.346		*Streptococcus agalactiae*
Streptococcus agalactiae	89,4	*Streptococcus agalactiae*	2.347		*Streptococcus agalactiae*
Streptococcus agalactiae	99,9	*Streptococcus agalactiae*	2.152		*Streptococcus agalactiae*
Streptococcus agalactiae	89,8	*Streptococcus agalactiae*	2.173		*Streptococcus agalactiae*
Streptococcus agalactiae	83,7	*Streptococcus agalactiae*	2.374		*Streptococcus agalactiae*
Streptococcus agalactiae	90,2	*Streptococcus agalactiae*	2.438		*Streptococcus agalactiae*
Streptococcus agalactiae	94,1	*Streptococcus agalactiae*	2.334		*Streptococcus agalactiae*
Streptococcus anginosus	86,6	*Streptococcus anginosus*	2.099		*Streptococcus anginosus*
Streptococcus anginosus	86,6	*Streptococcus constellatus*	2.159	*Streptococcus constellatus* [AF535184 (98.2%)]	*Streptococcus constellatus*
Streptococcus dysgalactiae	89,8	*Streptococcus dysgalactiae*	2.152		*Streptococcus dysgalactiae*

Streptococcus gordonii	86,9	*Streptococcus mitis*	2.223	*Streptococcus oralis* [AF535168 (98%)]	*Streptococcus oralis*
Streptococcus pyogenes	91,3	*Streptococcus pyogenes*	2.387		*Streptococcus pyogenes*
Streptococcus pyogenes	99,9	*Streptococcus pyogenes*	2.407		*Streptococcus pyogenes*
Streptococcus pyogenes	99,9	*Streptococcus pyogenes*	2.162		*Streptococcus pyogenes*
Streptococcus salivarius	84	*Streptococcus salivarius*	2.096		*Streptococcus salivarius*
